# Epidemiology of paediatric snakebites in Northeastern Nigeria: a retrospective single-center study

**DOI:** 10.1186/s12887-025-05910-3

**Published:** 2025-08-29

**Authors:** Nicholas Amani Hamman, Aashna Uppal, Ezra Garbeya Daniel, Nuhu Mohammed, Nyadah Nicholas, Abubakar Saidu Ballah, Nasiru Bappayo, Bello Abdulkadir, Bala Lawan, Joshua Abubakar Difa, Elon Warnnow Isaac

**Affiliations:** 1 Snakebite Treatment and Research Hospital, Kaltungo, Gombe State, Nigeria; 2 The Global Health Network, Centre for Global Health and Tropical Medicine, Nuffield Department of Medicine, University of Oxford, Oxford, UK; 3 Department of Paediatrics, Federal Teaching Hospital Gombe, Gombe State, Nigeria; 4 Gombe State Hospital Services Management Board, Gombe State, Nigeria; 5 Department of Community Medicine and Public Health, Gombe State University, Gombe State, Nigeria; 6 Department of Paediatrics, Gombe State University, Gombe State, Nigeria

**Keywords:** Snakebites, Paediatric patients, Nigeria, Epidemiology, Patient outcomes

## Abstract

**Background:**

Nigeria remains one of the highest burden bearers of snakebite envenoming in sub-Saharan Africa. In Northeastern Nigeria, where agricultural practice, livestock herding and outdoor play—especially during the dark hours—are common, children are frequently exposed to snakes. Due to the unique challenges posed by paediatric snakebite envenoming and the paucity of data on paediatric snakebites, there is need for local research on this subject. The study aims to bridge this knowledge gap by examining the characteristics and outcomes of paediatric snakebites in our setting.

**Methods:**

This was a retrospective study conducted at the Snakebite Treatment and Research Hospital (SBTRH) in Kaltungo, Northeastern Nigeria. Medical records of 723 patients aged 0 to 17 years treated at this facility between 1 January to 31 December 2024 were retrieved. Socio-demographic information and key clinical data were extracted from paper-based records and recorded in a Microsoft Excel document. The association between patient characteristics and likelihood of recovery without complications like amputation, debridement or death, was assessed using univariate and multivariable regression analyses.

**Results:**

There were 480 male patients (66%) and the median age of patients was 12 (range 1 to 17). Within the study period, snakebites in children were most common in April (*n* = 102, 14%). Nearly two-thirds of the participants (*n* = 468, 65%) took four hours or more to present to hospital after being bitten. Indeed, patients who took four hours or more to arrive to hospital were less likely to recover without complications (Unadjusted odds ratio (OR) = 0.24, 95% confidence interval (CI) = 0.12–0.43). A sub-analysis among patients who received antivenom revealed that antivenom dose, time to antivenom administration, and antivenom cost were all significantly associated with likelihood of recovery without complications.

**Conclusions:**

This study found that some patient characteristics may contribute to poor outcomes among paediatric snakebite patients in Northeastern Nigeria. It demonstrated the increased risk of complications among those presenting to hospital more than four hours after being bitten, those without timely antivenom administration, and those who paid for antivenom. We hereby recommend increased awareness and health education on early presentation after snakebites.

## Background

Snakebite envenoming remains a pressing public health challenge posing a significant threat, particularly in the tropical and subtropical regions of the world [1]. Numerous challenges affecting the tropics like poor road infrastructure, inadequate healthcare access, limited public awareness, environmental and socio-economic challenges converge to exacerbate the risk of envenoming and its devastating consequences [1, 2]. In 2017, the World Health Organization (WHO) reclassified snakebite envenoming as a neglected tropical disease, recognizing its disproportionate impact on health in low- and middle-income countries [3, 4]. Annually, an estimated 5.4 million individuals are bitten worldwide, with about 2.7 million cases resulting in envenoming and 138,880 fatalities [4]. Tragically, the incidence of amputations and other morbidities among survivors of snakebites is nearly three times the mortality figure [4, 5]. Nigeria, with an estimated 43,000 cases annually and 1,900 deaths, is one of the highest-burden countries, with carpet vipers– known for their haemotoxic venom– responsible for over 66% of bites [6].

Even though adults account for a substantial number of snakebite victims, children under the age of 15 years represent 30% of snakebite cases in Nigeria [7]. Due to their small size, faster venom spread, and unique physiology, children may experience more severe envenomation despite the availability of antivenom and supportive treatment [7–9]. This underscores the need for deliberate and improved research efforts in prevention and management. Agricultural activities, livestock herding, outdoor play, dipping hands into holes in search of small animals for consumption and flooding in Northeastern Nigeria further expose children to snakes [7, 10–12]. Their vulnerability is often compounded by limited healthcare access, poor knowledge of snakebite first aid, and management among healthcare workers in rural settings where these bites occur [13, 14]. This makes paediatric snakebite cases more challenging to manage [12, 14].

Children’s unique vulnerability can lead to more severe symptoms such as shock, renal failure, and in severe cases multi-organ failure [8, 15–18]. This is primarily due to the haemotoxic pathogenesis of viper venom, which disrupts blood clotting, damages the vascular endothelium, and can trigger systemic inflammation leading to multi-organ dysfunction [19]. Additionally, snakebite manifestations are harder to predict with various species causing different types of envenomation (e.g. haemotoxic, neurotoxic, or cytotoxic); as well, children are less likely to correctly identify snake species [20]. Despite growing awareness of snakebite in adults, paediatric cases remain underreported with a lack of data on risk factors-particularly in endemic areas [14]. This gap hinders the development of child-specific management protocols.

There is a notable lack of region-specific epidemiological data, especially from high-burden areas like Northeastern Nigeria, which limits the ability to develop targeted prevention and treatment strategies for paediatric populations. Therefore, this study aims to explore paediatric snakebite cases seen at the Snakebite Treatment and Research Hospital (SBTRH), Kaltungo, in Northeastern Nigeria, focusing on patient characteristics and their relation to envenoming outcomes. These findings will guide evidence-based management strategies and inform public health efforts to reduce snakebites in the region.

## Methods

This was a retrospective study conducted at SBTRH. This facility is located at the heart of Kaltungo Local Government Area (LGA), Gombe State, Northeastern Nigeria. It is a specialized hospital that has been focused on the management of snakebite envenoming for over five decades [21–23]. SBTRH provides both emergency and long-term care for victims of snakebite with a focus on treatment, training, clinical research, and public awareness campaigns. Interestingly, the hospital sees cases from all six states of Northeastern Nigeria, and from neighboring parts of the Northwestern and Northcentral regions of the country. Additionally, patients come from neighboring countries like Cameroon and the Republic of Chad.

In our sample, the size of which was not predetermined, we included all patients aged 0 to 17 (inclusive) presenting to SBTRH with snakebite from January through December 2024. We chose this age range as this is the most common age range for paediatric patients. We excluded patients with blank medical records and those who either absconded or left the institution against medical advice. A Microsoft Excel document was then used to extract data from paper-based clinical records containing patient information. We included the following information: month of snakebite occurrence, patient age, patient sex, patient state of origin, patient occupation, anatomical site of snakebite, species of snake, number of antivenom vials used, cost of antivenom (free vs. paid), number of hours between snakebite and presentation to hospital, number of hours between presentation to hospital and antivenom administration, and patient outcome (death, amputation, debridement, or recovery). Recovery was defined as the resolution of symptoms. To note, patient state of origin did not necessarily reflect exact distance to hospital.

The data was then cleaned and analyzed using R software (version 4.3.1). Two variables had missing data: snake species, as not every patient was able to identify the snake that bit them, and time between hospitalization and antivenom administration. We replaced blank values for snake species with “Unidentifiable” and left blank values for time between hospitalization and antivenom administration as is. We then explored which patient characteristics were associated with patient outcomes.

Patient characteristics were summarized, separately by a binary outcome (recovery versus amputation, debridement, and death). Because of low numbers, we grouped patients who experienced amputation, debridement or death. For categorical variables with cell counts greater or equal to five, a Chi-Squared test was used, and for categorical variables with cell counts less than five, the Fisher’s Exact test was used to evaluate differences in patient characteristics between treatment outcomes; differences were considered to be statistically significant if the corresponding test’s p-value was less than 0.05, as per convention.

Subsequently, we performed univariate and multivariable logistic regression analysis to explore associations between patient characteristics and likelihood of recovery without complications, compared to amputation, debridement, or death. We removed patients that were missing information and removed observations with cell counts equal to zero when separated by outcome. Multivariable regression analyses included all variables. Because the three antivenom variables (cost, dose, and time to antivenom) were colinear (namely, for patients that did not receive antivenom, these three variables were redundant), we performed three sets of multivariable regression analyses, each including one of the three antivenom variables. We reported both unadjusted and adjusted odds ratios, corresponding confidence intervals, and *p*-values from the univariate and multivariable regression models, respectively.

Ethical clearance was obtained from the Research and Ethics Committee, Gombe State Hospitals Services Management Board with reference number GS/HSMB/RES/S/05/VOL.59.

## Results

**Fig. 1:**
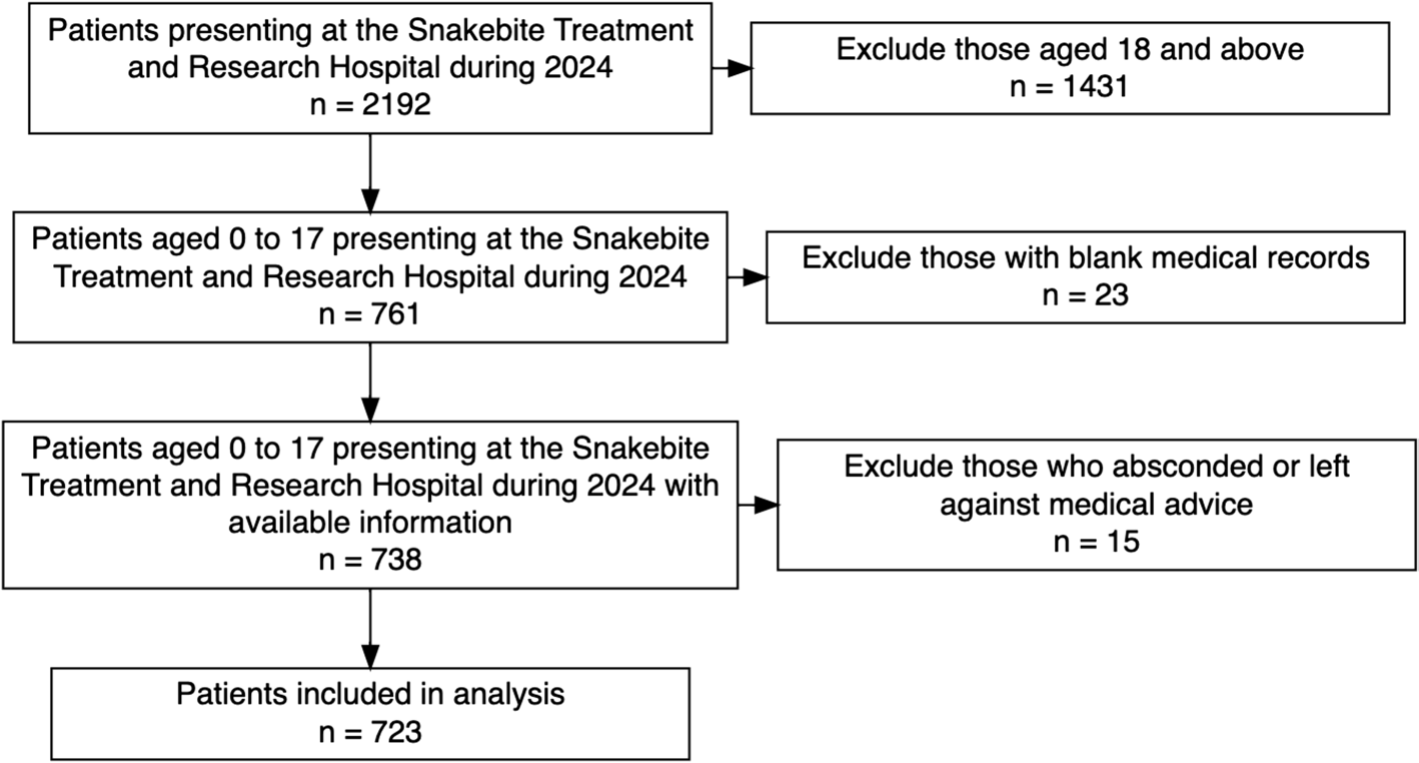
Flowchart of included patients

SBTRH managed 2,192 snakebite patients between 1 January and 31 December 2024, out of which 723 patients were included in analyses after applying exclusion criteria (Fig. 1).

Table 1 summarizes patients’ sociodemographic and clinical characteristics. Of the 723 paediatric patients’ records analysed, the median age was 12 (range 1 to 17), and children aged 10 to 14 years made up the majority (*n* = 300, 41%). Two-thirds of the patients were male (*n* = 480, 66%) and a minority were aged under five (*n* = 49, 7%). Nearly two-thirds took four hours or more to present to hospital after being bitten (*n* = 468, 65%), although more studies are needed to explore causes of delay. Further, most patients received a single dose of antivenom (*n* = 512, 71%), and most patients who received antivenom had it administered one hour or more after arriving at hospital (*n* = 491, 68%). Half of the patients resided within Gombe State (*n* = 364, 50%). The highest number of snakebites (*n* = 102, 14%) occurred in April, which is the start of the rainy season in several regions in Northeastern Nigeria. As these were paediatric patients, most either were either under care (*n* = 413, 57%) or were farmers (*n* = 143, 20%); “under care” refers to patients 16 years of age and under who are neither working nor in school. Snakebites predominantly occurred on the patients’ upper limbs (*n* = 434, 60%), and most bites were due to carpet vipers (*n* = 559, 77%). Finally, nearly all patients recovered without complications (*n* = 632, 87%). Of those that experienced complications (*n* = 91, 13%), there were 52 debridement surgeries, 21 deaths, and 18 amputations. Of the 21 patients that died, the age range was 2 to 17.

**Table 1:** Sociodemographic and clinical characteristics of the patients

**Variable**	**Overall**, *N* = 723^*1*^	**Patient outcome**		***p*-value**^*2*^
		**Amputation, debridement, or death**, *N* = 91^*1*^	**Recovery**, *N* = 632^*1*^	
Age	12 (8, 15)	12 (10, 15)	11 (8, 14)	0.006
Age Group				0.053
10 to 14	300	36 (12%)	264 (88%)	
5 to 9	189	19 (10%)	170 (90%)	
15 to 17	185	33 (18%)	152 (82%)	
0 to 4	49	3 (6.1%)	46 (94%)	
Sex				0.890
Male	480	61 (13%)	419 (87%)	
Female	243	30 (12%)	213 (88%)	
Hours Between Bite and Hospitalization				< 0.001
4 h or more^3^	468	79 (17%)	389 (83%)	
Less than 4 h	255	12 (4.7%)	243 (95%)	
Antivenom Dose (Number of Vials)				0.308
1	512	62 (12%)	450 (88%)	
0	112	12 (11%)	100 (89%)	
2 or more	99	17 (17%)	82 (83%)	
Hours Between Hospitalization and Antivenom Administration				0.018
1 h or more^3^	491	75 (15%)	416 (85%)	
No antivenom given	112	12 (11%)	100 (89%)	
Less than 1 h	87	4 (4.6%)	83 (95%)	
Not reported	33	0	33	
Antivenom Cost				< 0.001
Free	351	22 (6.3%)	329 (94%)	
Paid (for full or partial dose)	260	57 (22%)	203 (78%)	
No antivenom given	112	12 (11%)	100 (89%)	
State of Origin				
Gombe	364	41 (11%)	323 (89%)	
Taraba	122	8 (6.6%)	114 (93%)	
Adamawa	90	14 (16%)	76 (84%)	
Bauchi	67	11 (16%)	56 (84%)	
Borno	50	12 (24%)	38 (76%)	
Yobe	26	5 (19%)	21 (81%)	
Other^4^	4	0 (0%)	4 (100%)	
Month of Snakebite Occurrence				
April	102	5 (4.9%)	97 (95%)	
March	81	9 (11%)	72 (89%)	
August	73	15 (21%)	58 (79%)	
October	72	12 (17%)	60 (83%)	
July	67	16 (24%)	51 (76%)	
May	64	2 (3.1%)	62 (97%)	
June	59	3 (5.1%)	56 (95%)	
September	58	14 (24%)	44 (76%)	
February	47	3 (6.4%)	44 (94%)	
November	39	10 (26%)	29 (74%)	
January	31	1 (3.2%)	30 (97%)	
December	30	1 (3.3%)	29 (97%)	
Occupation				0.137
Under Care^5^	413	42 (10%)	371 (90%)	
Farmer	143	23 (16%)	120 (84%)	
Student	141	24 (17%)	117 (83%)	
House Wife	21	2 (9.5%)	19 (90%)	
Other^4^	5	0 (0%)	5 (100%)	
Site of Snakebite				0.300
Upper Limb	434	49 (11%)	385 (89%)	
Lower Limb	284	42 (15%)	242 (85%)	
Other^3^	5	0 (0%)	5 (100%)	
Snake Species				0.113
Carpet Viper	559	63 (11%)	496 (89%)	
Unidentifiable	157	27 (17%)	130 (83%)	
Other^4^	7	1 (14%)	6 (86%)	

^1^n (%)

^2^Fisher’s exact test (cell counts less than five); Pearson’s Chi-squared test (cell counts greater or equal to five). For some categories, p-values could not be calculated due to low cell counts

^3^The median number of hours between bite and hospitalization is 4, and the median number of hours between hospitalization and antivenom administration is 1

^4^Other values for state of origin are Cameroun and Plateau, for occupation are applicant and business, for site of snakebite are back and neck, and for snake species are cobra, night adder, and puff adder

^5^Under care refers to patients 16 years of age and under who are neither working nor in school

There were significant differences between patients who recovered without complications and those who did not. Four variables were significantly associated with patient outcomes: age (*p* = 0.006), hours between bite and hospitalization (*p* < 0.001), hours between hospitalization and antivenom administration (*p* = 0.018), and antivenom cost (*p* < 0.001).

Table 2 highlights the results of the univariate and multivariable regression analyses and demonstrates which patient characteristics were associated with likelihood of recovery. After removing patients that were missing information and cell counts that were equal to zero when separated by outcome, we were left with 690 patients for this analysis. In the univariate models, five variables were significantly associated with patient outcomes. First, patients who took four hours or more to arrive to hospital were less likely to recover without complications (Unadjusted odds ratio (OR) = 0.24, 95% confidence interval (CI) = 0.12–0.43) than those who arrived in less than four hours. Similarly, those who paid for antivenom were less likely to recover without complications (Unadjusted OR = 0.36, 95% CI = 0.28–0.68) than those who received no antivenom. Patients who arrived in July, August, September, and November were less likely to recover without complications compared to patients who arrived in January. Further, students were less likely to recover without complications than those under care (Unadjusted OR = 0.58, 95% CI = 0.34–1.01). Finally, patients whose state of origin was Borno were less likely to recover without complications (Unadjusted OR = 0.41, 95% CI 0.20–0.87).

**Table 2:** Likelihood of recovery without complications

Characteristic	Univariate models			Multivariable model 1			Multivariable model 2			Multivariable model 3		
	**Unadj. OR**^*1*^	**95% CI**^*1*^	***p*-value**	**Adj. OR**^*1*^	**95% CI**^*1*^	***p*-value**	**Adj. OR**^*1*^	**95% CI**^*1*^	***p*-value**	**Adj. OR**^*1*^	**95% CI**^*1*^	***p*-value**
Age Group												
0 to 4	—	—	—	—	—	—	—	—	—	—	—	—
5 to 9	0.56	0.13, 1.74	0.366	0.95	0.19, 3.52	0.944	0.88	0.17, 3.34	0.865	0.81	0.17, 2.97	0.772
10 to 14	0.48	0.11, 1.41	0.240	1.16	0.23, 4.25	0.836	1.25	0.24, 4.72	0.767	1.03	0.21, 3.72	0.966
15 to 17	0.30	0.07, 0.90	0.057	0.66	0.12, 2.86	0.603	0.76	0.13, 3.41	0.733	0.59	0.11, 2.49	0.499
Sex												
Female	—	—	—	—	—	—	—	—	—	—	—	—
Male	0.96	0.60, 1.53	0.877	0.87	0.48, 1.56	0.654	1.02	0.56, 1.85	0.937	0.87	0.48, 1.55	0.642
Hours Between Bite and Hospitalization												
$<$4 h	—	—	—	—	—	—	—	—	—	—	—	—
$\ge$4 h	0.24	0.12, 0.43	< 0.001	0.19	0.08, 0.41	< 0.001	0.20	0.09, 0.43	< 0.001	0.20	0.09, 0.43	< 0.001
Antivenom Dose (Number of Vials)												
0	—	—	—	—	—	—	—	—	—	—	—	—
1	0.81	0.40, 1.51	0.528	0.42	0.19, 0.87	0.026						
2 or more	0.56	0.25, 1.24	0.156	0.14	0.05, 0.38	< 0.001						
Hours Between Hospitalization and Antivenom Administration												
No antivenom	—	—	—				—	—	—			
$<$1 h	2.54	0.85, 9.36	0.118				2.42	0.71, 9.80	0.176			
$\ge$1 h	0.66	0.33, 1.22	0.211				0.27	0.12, 0.56	< 0.001			
Antivenom Cost												
No antivenom	—	—	—							—	—	—
Free	1.78	0.83, 3.67	0.126							0.41	0.06, 1.61	0.263
Paid	0.36	0.28, 0.68	0.003							0.36	0.15, 0.80	0.016
State of Origin												
Gombe	—	—	—	—	—	—	—	—	—	—	—	—
Adamawa	0.69	0.36, 1.37	0.267	1.07	0.48, 2.42	0.875	1.18	0.53, 2.70	0.692	1.08	0.50, 2.43	0.846
Bauchi	0.63	0.31, 1.36	0.213	1.17	0.48, 2.94	0.738	0.84	0.34, 2.15	0.713	0.99	0.42, 2.47	0.988
Borno	0.41	0.20, 0.87	0.016	0.85	0.36, 2.08	0.710	0.68	0.28, 1.72	0.404	0.82	0.34, 2.03	0.666
Taraba	1.82	0.87, 4.29	0.138	3.20	1.33, 8.48	0.013	3.22	1.33, 8.51	0.013	2.85	1.20, 7.38	0.022
Yobe	0.52	0.20, 1.62	0.211	1.22	0.38, 4.53	0.747	1.08	0.32, 4.21	0.906	1.18	0.37, 4.35	0.786
Month of Snakebite Occurrence												
January	—	—	—	—	—	—	—	—	—	—	—	—
February	0.49	0.02, 4.08	0.550	0.20	0.01, 1.82	0.189	0.43	0.02, 3.70	0.479	0.27	0.01, 2.37	0.277
March	0.26	0.01, 1.48	0.212	0.16	0.01, 1.07	0.110	0.28	0.01, 1.71	0.247	0.19	0.01, 1.17	0.133
April	0.66	0.03, 4.28	0.705	0.37	0.02, 2.73	0.396	0.74	0.04, 5.11	0.791	0.47	0.02, 3.30	0.508
May	1.05	0.05, 11.4	0.968	0.66	0.03, 8.07	0.751	1.39	0.06, 15.9	0.795	0.89	0.04, 10.3	0.925
June	0.64	0.03, 5.28	0.708	0.33	0.01, 3.02	0.366	0.66	0.03, 5.74	0.733	0.43	0.02, 3.84	0.490
July	0.06	0.00, 0.30	0.007	0.02	0.00, 0.16	0.001	0.05	0.00, 0.31	0.007	0.04	0.00, 0.44	0.021
August	0.11	0.01, 0.58	0.036	0.05	0.00, 0.29	0.007	0.08	0.00, 0.44	0.018	0.08	0.00, 0.85	0.064
September	0.11	0.01, 0.57	0.035	0.06	0.00, 0.37	0.012	0.09	0.00, 0.55	0.030	0.09	0.00, 1.04	0.087
October	0.17	0.01, 0.94	0.099	0.09	0.00, 0.59	0.035	0.16	0.01, 0.96	0.097	0.15	0.01, 1.77	0.179
November	0.10	0.01, 0.57	0.033	0.04	0.00, 0.27	0.005	0.04	0.00, 0.29	0.006	0.06	0.00, 0.75	0.052
December	1.00	0.04, 26.1	1.000	0.99	0.03, 28.1	0.993	0.53	0.02, 14.9	0.668	0.76	0.03, 21.9	0.855
Occupation												
Under Care	—	—	—	—	—	—	—	—	—	—	—	—
Student	0.58	0.34, 1.01	0.049	0.54	0.24, 1.20	0.126	0.48	0.21, 1.07	0.072	0.49	0.23, 1.09	0.078
Farmer	0.60	0.35, 1.06	0.072	0.85	0.37, 2.01	0.711	0.77	0.33, 1.84	0.545	0.86	0.38, 2.00	0.715
House Wife	1.11	0.30, 7.11	0.896	1.89	0.31, 17.1	0.522	1.60	0.25, 14.9	0.645	1.70	0.28, 15.3	0.591
Site of Snakebite												
Upper Limb	—	—	—	—	—	—	—	—	—	—	—	—
Lower Limb	0.70	0.45, 1.10	0.118	1.04	0.58, 1.87	0.893	0.99	0.55, 1.81	0.976	1.02	0.57, 1.83	0.949
Snake Species												
Carpet Viper	—	—	—	—	—	—	—	—	—	—	—	—
Unidentifiable	0.63	0.39, 1.05	0.070	0.43	0.24, 0.79	0.006	0.43	0.23, 0.80	0.007	0.44	0.24, 0.80	0.007
Other	0.83	0.14, 15.8	0.863	0.31	0.03, 7.37	0.367	0.31	0.02, 9.28	0.423	0.24	0.02, 6.33	0.295

^*1*^*Unadj. OR* Unadjusted Odds Ratio, *Adj. OR* Adjusted Odds Ratio, *CI* Confidence Interval

In multivariable models, some variables remained significantly associated with patient outcome, while others did not. Patients who took four hours or more to arrive at hospital, and patients who presented between July and November (excluding October in some cases), remained less likely to recover without complications. On the other hand, patient occupation was no longer significantly associated with likelihood of recovery in all multivariable models. There were two variables that were not significantly associated with likelihood of recovery in univariate models, but were significantly associated with likelihood of recovery in multivariable models. First, patients presenting from Taraba were more likely to recover without complications compared to patients from other states (Model 1 Adjusted OR = 3.20, 95% CI = 1.33–8.48; Model 2 Adjusted OR = 3.22, 95% CI = 1.33–8.51; Model 3 Adjusted OR = 2.85, 95% CI 1.20–7.38). Second, patients who were unable to identify snake species were less likely to recover without complications than those bitten by carpet vipers (Model 1 Adjusted OR = 0.43, 95% CI = 0.24–0.79; Model 2 Adjusted OR = 0.43, 95% CI = 0.23–0.80; Model 3 Adjusted OR = 0.44, 95% CI 0.24–0.80).

The three antivenom variables were also significantly associated with likelihood of recovery without complications. Patients who received a single dose (Adjusted OR = 0.42, 95% CI = 0.19–0.87) or multiple doses (Adjusted OR = 0.14, 95% CI = 0.05–0.38) of antivenom were less likely to recover without complications than those who received no antivenom. As well, patients who were administered antivenom one hour or more after presentation to hospital were less likely to recover without complications compared to patients who received no antivenom (Adjusted OR = 0.27, 95% CI = 0.12–0.56). Finally, patients who paid for antivenom were less likely to recover compared to patients who received no antivenom (Adjusted OR = 0.36, 95% CI = 0.15–0.80). Of note, the observation that patients who did not receive antivenom had better outcomes likely reflects a severity bias.

Table 3 illustrates the results of a sub-analysis which takes a closer look at the three antivenom variables, for 567 patients who received antivenom. Compared to those who received a single vial of antivenom, those who received two or more vials were less likely to recover without complications (Adjusted OR = 0.46, 95% CI = 0.24–0.92), potentially because antivenom dosage often corresponds to symptom severity. As well, compared to patients who received antivenom less than one hour after hospitalization, patients who received antivenom after one or more hours were less likely to recover without complications (Adjusted OR = 0.12, 95% CI = 0.04–0.31). Finally, patients who paid for antivenom were less likely to recover without complications (Adjusted OR = 0.12, 95% CI = 0.07–0.22) compared to patients that received antivenom for free.

**Table 3:** Likelihood of recovery without complications among those who received antivenom

**Characteristic**	**Adjusted OR**^*1*^	**95% CI**^*1*^	*p***-value**
Antivenom Dose (Number of Vials)			
1	—	—	—
2 or more	0.46	0.24, 0.92	0.024
Hours Between Hospitalization and Antivenom Administration			
Less than 1 h	—	—	—
1 h or more	0.12	0.04, 0.31	< 0.001
Antivenom Cost			
Free	—	—	—
Paid (for Full or Partial Dose)	0.12	0.07, 0.22	< 0.001

^1^*OR* Odds Ratio, *CI* Confidence Interval

## Discussion

This pilot study, part of a broader effort to digitize five years of paediatric snakebite data at SBTRH, analyses 723 cases treated from January through December 2024. It offers insights into the epidemiology, outcomes, and recovery factors for children in this high-risk region. To our knowledge, this is the largest single-site study of its kind. The analysis includes demographic data, time to presentation, snakebite species, antivenom use, and patient outcomes, providing a comprehensive view of factors influencing recovery.

A key finding of the study is the male predominance among paediatric snakebite victims, with 66% of the cases being male. This is consistent with many studies in sub-Saharan Africa [7, 23–25] where male gender, particularly in rural and agricultural communities, are at greater risk of encountering snakes than female. This may be due to greater exposure to outdoor environments, because males are more likely to engage in activities such as farming, livestock tending, outdoor play, errands during dark hours and other outdoor activities, which put them in closer proximity to venomous snakes.

This study, which was based on records of patients who presented throughout the year, found that snakebites were most common in the rainy season (April to October), highlighting a seasonal variation in snakebite incidence. Snakebite incidence has been well documented in both tropical and subtropical regions to be linked to rainfall patterns [23, 26–28]. This seasonal trend aligns with similar findings from studies in Eastern Nepal, Sri Lanka and Northwestern Nigeria, where rainy seasons coincide with increased agricultural activity and heightened human-snake interaction, particularly among children [29–31]. These comparisons enhance the relevance and generalizability of our findings to other tropical low-resource settings. This seasonal peak implies that targeted interventions during these months could be particularly effective. These may include community education emphasizing the importance of protective hand- and footwear, and avoiding areas known to be predisposed to the presence of snakes, such as holes and bushes.

This study also noted that about 65% of patients arrived four hours or more after being bitten, indicating a significant delay in presentation. This delay may be due to a combination of contextual barriers, including limited access to healthcare facilities, transportation challenges in rural areas, delays at primary health centres prior to referral, and general unawareness of the urgency required in responding to snakebites. Similar barriers have been documented in studies from Kenya and India, where factors such as geographical distance, financial constraints, and poor infrastructure hinder timely medical access for snakebite victims [32, 33]. However, this delay is a key concern because snakebite envenomation can progress rapidly, leading to complications when definitive intervention is not applied as early as possible. Interestingly, the study found that patients who took four or more hours to arrive at the hospital after being bitten were less likely to recover without complications compared to those who arrived within four hours (Unadjusted OR 0.24, 95% CI 0.12 to 0.43). This suggests a significant trend toward improved recovery with earlier hospital arrival, reinforcing the well-established association between delayed medical treatment and poor outcomes for snakebite victims, particularly in rural areas with limited healthcare access [11–13, 21, 34]. To address this, targeted public health interventions are recommended, including community education on the urgency of seeking prompt care after a snakebite and the development of a rapid referral and transport system in rural areas to minimize delays in accessing treatment.

Unexpectedly, the study also found that hands were the most commonly affected site (60%) which is a significant departure from global, regional and even local studies [35–39], which consistently report that lower extremities are more frequently affected. This unusual pattern is noteworthy and may reflect specific regional or cultural practices unique to Northeastern Nigeria. For example, the children frequently use their hands during farming, foraging, or when probing into holes and bushes—behaviours that increase of upper limb bites. The only comparable finding was observed in parts of Southern Croatia where similar rural and subsistence lifestyles exist [40]. This parallel further strengthens the argument that local behaviours rather than random chance are driving the observed bite patterns in this region. Interestingly, hands are typically the first point of contact when a child encounters animals like snakes, whether while playing, working, or performing other tasks [7, 8, 41–43]. Such a finding challenges conventional assumptions and calls for further research into the specific local dynamics and regional practices that could influence snakebite patterns. This could be valuable for prospective studies, which we intend to undertake to confirm our findings.

The study found that most patients (71%) received a single vial of antivenom, which is consistent with a trial conducted at our institution, which showed that a single vial of Echitab polyvalent antivenom– the mainstay of treatment in the facility– was as effective and safe as two vials in treating moderate carpet viper envenomation in children [44]. Significantly, patients who received a single vial were more likely to recover without complications (*p* = 0.024). However, this association may be confounded by the fact that severely envenomed patients often require higher doses of antivenom and are at greater risk of severe outcomes including amputation, debridement, or death. As a result, the number of vials administered was excluded from the regression analysis to avoid potential reverse causality.

A majority of the cases (77%) were attributed to the carpet viper which has been documented to be responsible for most snakebites in this region [7, 23, 45, 46]. This further emphasizes the need for effective and affordable antivenom targeting these species, given that its venom is primarily haematoxic and can lead to severe coagulopathy and tissue damage [9, 41]. Additionally, understanding the pathogenesis of envenomation, particularly the haematoxic effect of Viperidae species, could provide further insight into the mechanisms driving such severe outcomes [19]. Moreover, this study did not examine the potential effect of this specie on renal function and bleeding disorders due to lack of available data, which remains an important area for future research. However, 21% of cases in our study were bitten by unidentifiable snakes, underscoring the challenges of accurately identifying snakes in emergency settings, particularly in rural areas and among paediatric patients. Indeed, patients in our study who were unable to identify the species of snake that bit them were less likely to recover without complications than those bitten by carpet vipers (Model 1 Adjusted OR = 0.43, 95% CI = 0.24–0.79; Model 2 Adjusted OR = 0.43, 95% CI = 0.23–0.80; Model 3 Adjusted OR = 0.44, 95% CI 0.24–0.80). Accordingly, snake identification is one of the first priorities in snakebite management; accurate identification is essential because it allows healthcare providers to predict symptoms, select the correct antivenom, and improve patient outcomes.

A notable finding of this study is that patients who received antivenom after one hour of hospitalization were significantly less likely to recover compared to those who received antivenom within one hour of hospitalization (Adjusted OR = 0.12, 95% CI = 0.04–0.31). Additionally, patients who paid for antivenom were less likely to recover without complications compared to those who received antivenom for free (Adjusted OR = 0.36, 95% CI = 0.15–0.80), which may reflect broader issues related to healthcare access and affordability. This may also be related to the finding that patients who presented during months without freely available antivenom (predominantly in the latter half of the year) were also less likely to recover without complications, as noted in Table 2. Together, these findings highlight the importance of both timely treatment and the removal of financial barriers in improving outcomes for snakebite victims, particularly in low-resource settings.

Despite its unique and valuable findings, this study is not without limitations. The retrospective design, which relied on paper-based records, might have introduced biases related to data accuracy and completeness. Additionally, while the study was conducted at a single-centre hospital, it serves as both a primary and a referral center for all states of Northeastern Nigeria, as well as parts of Northwestern Nigeria, North-Central Nigeria, Cameroon and Chad. This broad catchment area partially mitigates concerns about the generalizability of the findings. Although most cases were primary rather than referred, this broad catchment area could also lead to some degree of overrepresentation of more severe cases, which could potentially skew outcomes. Furthermore, data on the type of treatment patients received prior to hospital admission and the exact distance from the patient’s location to the hospital were not consistently recorded, limiting our ability to account for pre-hospital care and the impact of travel time on outcomes. Similarly, we were not able to report complications such as acute kidney injury (AKI), coagulopathies and bleeding disorders due to a lack of available data.

To build on these results and gain a more comprehensive understanding of paediatric snakebite epidemiology and outcomes, future research should aim to conduct single-centre or multi-centre prospective studies with standardized data collection protocols. Such studies would offer a more detailed and representative picture of the factors influencing outcomes in paediatric snakebite envenomation across diverse settings in the region.

## Conclusions

This study provided an analysis of 723 paediatric snakebite cases from a specialised facility in Northeastern Nigeria, exploring key characteristics that contributed to patient outcomes in the region. A notable finding was predominance of upper extremity bites which challenges conventional global trends and suggests that specific regional or behavioural factors may influence the patterns of bites. Additionally, the study found that carpet viper was the most common specie involved in these incidents, highlighting the need for effective species-targeted antivenom in the region. The study demonstrated that early administration of antivenom and removal of financial barriers are critical for improving patient outcomes. These findings underscore the importance of tailoring public health interventions and clinical protocols to address the unique patterns of snakebites in this area. The results also point to the need for further research to better understand the local dynamics, improve prevention strategies, and enhance treatment outcomes for paediatric snakebite victims. This study serves as an important step toward improving healthcare responses to snakebites in Northeastern Nigeria and similar regions, ultimately contributing to the reduction of snakebite morbidity and mortality.

## Data Availability

The data that support the findings of this study are not openly available due to reasons of sensitivity and are available from the corresponding author upon reasonable request. Data are located in controlled access data storage at the Snakebite Treatment and Research Hospital, Kaltungo.

## Abbreviations

AKI: Acute Kidney Injury
CI: Confidence Interval
LGA: Local Government Area
OR: Odds Ratio
SBTRH: Snakebite Treatment and Research Hospital
WHO: World Health Organization
